# Thinned-Young Apple Polyphenols Inhibit Halitosis-Related Bacteria Through Damage to the Cell Membrane

**DOI:** 10.3389/fmicb.2021.745100

**Published:** 2022-02-23

**Authors:** Ting Liu, Hailiang Shen, Furong Wang, Xueru Zhou, Pengtao Zhao, Yali Yang, Yurong Guo

**Affiliations:** ^1^College of Food Engineering and Nutritional Science, Shaanxi Normal University, Xi’an, China; ^2^National Research and Development Center of Apple Processing Technology, Xi’an, China; ^3^Citrus Research Institute, Southwest University, Chongqing, China; ^4^Citrus Research Institute, Chinese Academy of Agricultural Sciences, Chongqing, China

**Keywords:** Thinned-young apple polyphenols, halitosis, antibacterial mechanism, cell membrane, membrane potential

## Abstract

The thinned young apple is a by-product and is generally discarded in the orchard during fruit thinning. The polyphenol content of thinned young apples is about 10 times more than that of ripe apples. In our study, the antibacterial effect of thinned young apple polyphenols (YAP) on the halitosis-related bacteria including *Porphyromonas gingivalis*, *Prevotella intermedius*, and *Fusobacterium nucleatum* was investigated. The minimum inhibitory concentrations of YAP against *P. gingivalis*, *P. intermedia*, and *F. nucleatum* were 8.0, 8.0, and 12.0 mg/ml, while the minimum bactericidal concentrations were 10.0, 10.0, and 14.0 mg/ml, respectively. The scanning electron microscopy and transmission electron microscopy analyses showed that after YAP treatment, the membrane surface of halitosis-related bacterial cells was coarse and the cell wall and membrane were separated and eventually ruptured. The integrity of the cell membrane was determined by flow cytometry, indicating that the cells with the integrity membrane significantly reduced as the YAP concentration treatment increased. The release of proteins and nucleic acids into the cell suspension significantly increased, and the membrane potential reduced after the YAP treatment. This research illustrated the antibacterial mechanism of YAP against halitosis-related bacteria and provided a scientific basis of utilizing the polyphenols from the discarded thinned young apples.

## Introduction

Apples are rich in lots of nutrients, such as vitamin, fiber, pectin, and polyphenol which are good for the health. The major apple producers around the world are China, Italy, France, and United States (Nicolas et al., 1994). The total apple yield all over the world in 2014 was 84.56 × 10^6^ and 40.92 × 10^6^ tons in China, accounting for 48.39% of the total yield (Li et al., 2018). However, in order to improve the color, size, and quality of apples at harvest, the extra small thinned young apples should be removed from the apple tree after flowering (Miller and Rice-Evans, 1997). In China, about 1.9 million tons of thinned young apples are abandoned every year (Dou et al., 2015). These thinned young apples are usually directly discarded on the orchard grounds and may become a good energy source for the growth of microorganisms, which could increase the risk of fruit diseases and result in a significant reduction in the quality and yield of fruits (Hou et al., 2019). In recent years, some research has focused on the functional properties of thinned young apples (Chen W. et al., 2015; Yuan et al., 2016; Chen et al., 2017). Zhang et al. (2017) found that the polyphenols of thinned young apples have significant antibacterial activity against *Staphylococcus* and *Bacillus anthracis*. Nisar et al. (2019) reported that pectin films incorporated with young apple polyphenols could efficiently inhibit the growth of *Staphylococcus aureus*, *Escherichia coli*, and *Listeria monocytogenes*.

Halitosis is defined as an unpleasant odor caused by the catabolism of bacterial coverage of the tongue, periodontal disease, and other systemic diseases (Joda and Olukoju, 2013), but in halitosis, an incidence of 80–90% are caused by bacteria in the oral cavity (Sanz et al., 2001; Armstrong et al., 2010). *Porphyromonas gingivalis*, *Prevotella intermedius*, and *Fusobacterium nucleatum* are considered to be the main bacteria inducing halitosis (Wang et al., 2002; Lau et al., 2019). The sulfur volatiles such as hydrogen sulfide (H_2_S) are mainly in the halitosis odor, which are generated by sulfur amino acids such as cystine, cysteine, and methionine. The chemical solutions including chlorhexidine, triclosan, and cetylpyridinium chloride are usually used to inhibit the halitosis odor, but they may induce side effects such as bacterial resistance and urticaria (Peruzzo et al., 2007; Cortelli et al., 2008). In recent years, many studies pay attention to identifying the safe and natural antibiotic properties such as essential oil, saponin, and phenolic compounds of fruits and vegetables for inhibiting the bacteria-related halitosis odor (Nijole et al., 2018; Sun et al., 2019; Lagha et al., 2020). However, there are rare studies about the antibacterial activity of phenolic compounds of thinned young apples against the bacteria-related halitosis.

In this study, the antibacterial effect and mechanism of young apple polyphenols (YAP) against *P. gingivalis*, *P. intermedius*, and *F. nucleatum* were investigated. This study aimed to identify the natural and safe phenolic compounds of thinned young apples for the suppression of bacteria-related halitosis, which provided a new environmental way of using thinned young apples.

## Materials and Methods

### Materials and Chemicals

The apple cultivar ‘Fuji’ was obtained at the Baishui Apple Test Station of Northwest Agriculture and Forestry University in Shaanxi province (China). The thinned young apples were collected 35 days after blossom and stored at –80°C. All polyphenol standards used for high-performance liquid chromatography (HPLC) analysis were purchased from Yuanye Biotechnology (Shanghai, China). *P. intermedia*, *P. gingivalis*, and *F. nucleatum* were obtained from Bena Culture Collection (BNCC) (Beijing, China).

### Extraction, Purification, and Determination of Thinned Young Apple Polyphenols

The YAP were extracted and purification according to our previous method (Gong et al., 2020). Briefly, the thinned young apples were crashed into 3–4-mm particles. The crude polyphenols were extracted with 70% alcohol at 65°C for 3 h. The extract was filtered with a Buchner funnel and concentrated in a rotary evaporator (OSB-2100, Shanghai Ailang Instrument Factory, China). Then, the solution was centrifuged at 3,500 × *g* for 20 min and the supernatant was collected and eluted by an X-5 macroporous resin. Subsequently, the polyphenol extract was concentrated and lyophilized to obtain the polyphenol powder of ‘Fuji’ thinned young apples. According to the Folin–Ciocalteu method, the total polyphenol content in YAP was determined and expressed as gallic acid equivalent (mg GAE/g) (Chen L. Y. et al., 2015). The individual phenol compounds were analyzed by HPLC according to our previous method (Wang et al., 2019).

### Antibacterial Activity

*Porphyromonas gingivalis*, *F. nucleatum*, and *P. intermedia* were used in this experiment. The minimum inhibitory concentration (MIC) and the minimum bactericidal concentration (MBC) of YAP were determined according to the methods reported by Wang et al. (2013). Briefly, bacterial cells were cultured in BHI liquid medium which was added with Vitamin K_3_, yeast extracted, and Hemin. The bacterial cell concentration was adjusted at 1 × 10^5^ colony-forming units per ml (CFU/ml). Then, the different concentrations of YPA (16, 14, 12, 10, 8, 4, and 2 mg/ml) were added in the test samples with agent-free broth as the blank. All the samples were incubated at 37°C for 48 h. MIC was defined as the lowest antibacterial concentration that inhibited bacterial growth, as shown by the absence of turbidity. The MBC was analyzed by inoculating 10 μl of medium from each of the MIC test that showed no turbidity onto BHI agar plates and incubation at 37°C for 48 h. The MBC values were defined as the lowest concentrations of antibacterial agents where there was no bacterial growth on the plates.

### Microstructure Analysis

Bacteria in this study were cultured in BHI liquid medium with different concentrations of YPA (control, MIC, and MBC) and incubated at 37°C for 24 h. Then each culture was harvested by centrifugation at 3,000 × *g* for 10 min. The samples were prepared according to the method of Wang et al. (2018) and then were observed and photographed by scanning electron microscopy (SEM; Quanta 200, FEI Co., Hillsboro, OR, United States).

The intracellular microstructure was observed and photographed by transmission electron microscope (TEM; H-7650, Hitachi Co., Tokyo, Japan). The preparation of TEM samples was performed according to the method of Wang et al. (2018). The pellets were fixed in 2.5% (v/v) glutaraldehyde for 90 min and washed three times by 0.1 M phosphate buffer (pH 7.2). The cells of each group were fixed in 1% osmic acid for 2 h at room temperature. Then, the bacterial cells were dehydrated and infiltrated into acetone and epoxy resin. The samples were embedded, polymerized, and sectioned.

### Cell Membrane Integrity Analysis

According to the previously reported method (Zhou et al., 2020), the bacterial cells stained by dye propidium iodide (PI) were used to evaluate the cell membrane integrity of *P. gingivalis*, *F. nucleatum*, and *P. intermedia via* flow cytometry. The cells used for cell membrane integrity analysis was prepared by the same method as the SEM analysis. After incubation and centrifugation, the bacterial cells were washed three times by 0.1 M phosphate buffer (pH 7.2) and the pellets were resuspended in 0.1 M phosphate buffer. 1 ml of cell suspension was stained with 3 μl of PI (5 mM) for 20 min at 37°C in the dark. The fluorescence intensity was detected by the BD Accuri C6 flow cytometer (Becton Dickinson, United States), and the NovoExpress software was used for data analysis.

### The Release of Proteins and Nucleic Acids

The changes in DNA content outside the cell membrane were graphed with the optical density and the corresponding time as the ordinate and abscissa, respectively. Specifically, bacterial cells were cultured in BHI liquid medium combined with different concentrations of YPA (control, MIC, and MBC) at 37°C for 24 h. Every 4 h, the suspensions were collected and centrifuged at 5,000 × *g* for 10 min; the supernatants were collected and diluted with 0.1 M phosphate buffer. Then, the optical density was determined with a microplate reader (Multiskan GO, Molecular Devices, Sunnyvale, CA, United States) at 260 nm. The corrections were carried out for the optical density at 260 nm of suspensions with PBS (0.1 M, pH 7.4) containing the same concentrations of YAP. In addition, the suspension was collected to determine the protein concentration according to Bradford’s method (Bradford, 1976).

### Membrane Potential Determination

The cell suspensions (approximately 1 × 10^7^ CFU/ml) were combined with different concentrations of YPA (control, MIC, and MBC) and incubated at 37°C for 8 h. The suspensions were washed three times with 0.1 M phosphate buffer (pH 7.2) and mixed with 2 μg/ml of rhodamine 123. Then, the samples were washed three times again, and the pellets were resuspended in PBS for 30 min in the dark. The cell suspensions were a 96-well microplate and detected by a microplate reader (Multiskan Go, Molecular Devices, Sunnyvale, CA, United States) (Comas and Vives-Rego, 1997). The data were expressed as median fluorescent intensity (MFI).

### Statistical Analysis

All experiments were done in triplicate. The data were analyzed by using Origin 8.0 (OriginLab Co., Northampton, MA, United States) and SPSS software 24.0 (SPSS Inc., Chicago, IL, United States) and expressed as the average ± standard deviations. Duncan’s multiple-range test with 95% confidential level was used to access the difference between the average values. *p* < 0.05 indicated the significant difference between variables. Principle component analysis (PCA) determined the relationships among the variables by the STAT-ITCF Statistical software (Bordeaux, France).

## Results

### Chemical Characteristics of YAP

**FIGURE 1:**
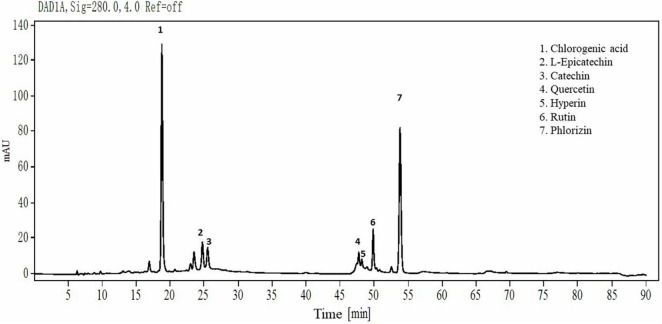
HPLC chromatograms of YAP. YAP was 70% ethanol-eluted fractions from thinned young apples.

As Figure 1 shows, the main individual phenols in thinned young apples are chlorogenic acid, L-epicatechin, catechin, quercetin, hyperin, rutin, and phlorizin. Their contents were 37, 5.28, 4.37, 6.02, 2.59, 6, and 29%, respectively. The contents of phlorizin and chlorogenic acid were the highest, accounting for 66% of the total phenolic content.

### Antibacterial Activity of YAP

The antibacterial activity of YAP was evaluated by MIC and MBC. The MIC values of *P. gingivalis*, *F. nucleatum*, and *P. intermedia* were 8.0, 8.0, and 12.0 mg/ml, respectively, while the MBC values were 10.0, 10.0, and 14.0 mg/ml, respectively. Among the three bacteria, *P. gingivalis* and *F. nucleatum* were similarly susceptible to the YAP, with the lower MIC and MBC.

### Effect of YAP on the Microstructure of Bacterial Cells

**FIGURE 2:**
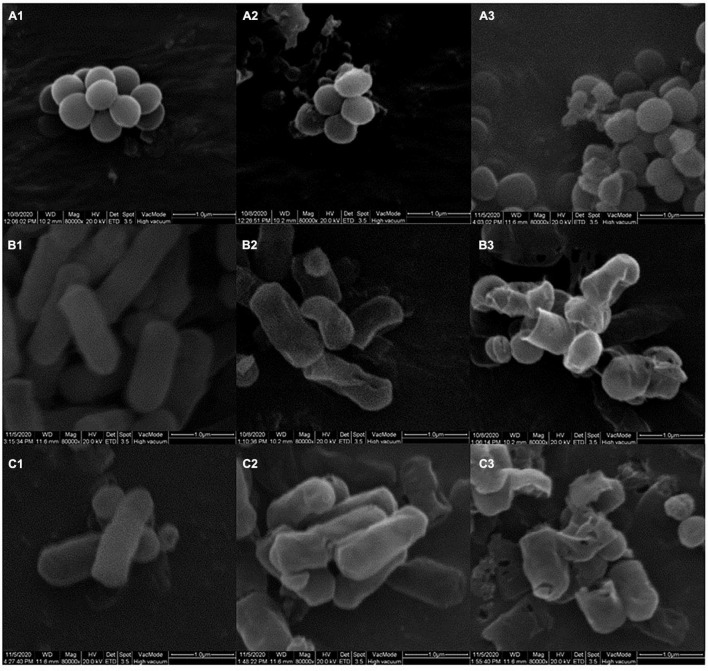
The outer wall structure changes of bacterial cells treated with different concentrations of YAP. **(A1)** *P. intermedia* with untreated, as control; **(A2)** *P. intermedia* treated with MIC; **(A3)** *P. intermedia* treated with MBC; **(B1)** *P. gingivalis* with untreated, as control; **(B2)** *P. gingivalis* treated with MIC; **(B3)** *P. gingivalis* treated with MBC; **(C1)** *F. nucleatum* with untreated, as control; **(C2)** *F. nucleatum* treated with MIC; **(C3)** *F. nucleatum* treated with MBC. MIC, minimum inhibition concentration; MBC, minimum bactericide concentration.

In order to investigate the effects of YAP on the outer wall structure of bacterial cells, SEM was used to analyze the wall structure changes of bacterial cells after the different concentrations of YAP (control, MIC, and MBC) treatment. As shown in Figure 2, untreated *P. intermedia* cells (Figure 2A1) showed the typical globe-shaped morphology, while *P. gingivalis* (Figure 2B1) and *F. nucleatum* (Figure 2C1) cells showed the typical rod-shaped morphology. After treatment with YAP at the MIC level, the bacterial cells (Figures 2A2,B2,C2) showed severe outer wall morphological changes. Additionally, the surface of bacterial cells treated with YAP at the MBC level (Figures 2A3,B3,C3) showed serious wrinkles and pores, and some cells were even broken, which indicated that the outer wall morphological changes were more serious compared to the bacterial cells treated with YAP at the MIC level. The observations of SEM suggested that the cell surface of *P. intermedia*, *P. gingivalis*, and *F. nucleatum* could be damaged by the YAP, which could affect the proliferation of bacterial cells and may lead to cell death. However, it was unclear whether the morphological changes of bacterial cells induced by YAP could lead to the penetration of the bacterial envelope, which was due to the fact that the YAP may pierce through the bacterial envelope and damage the cell barrier between the cytosol and extracellular environment. Thus, TEM analysis was further used to analyze this phenomenon.

**FIGURE 3:**
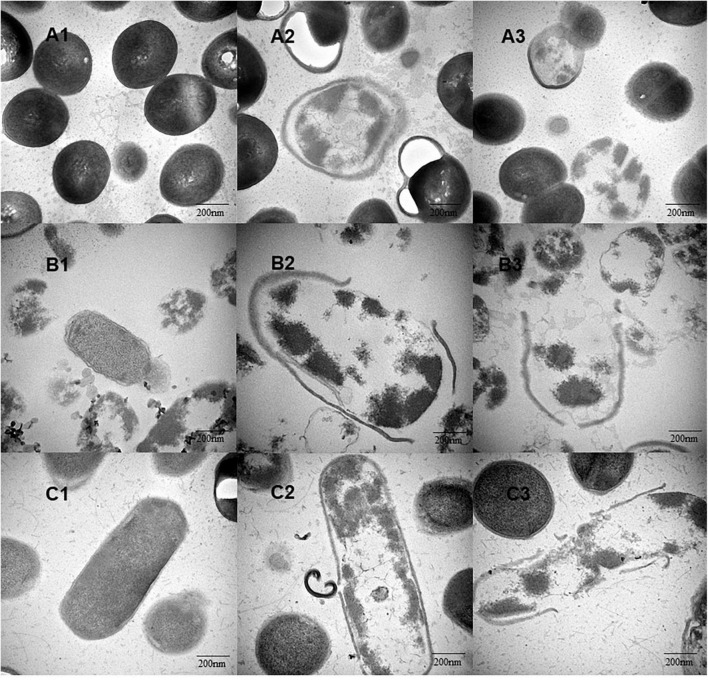
The intracellular microstructure changes of bacterial cells treated with different concentrations of YAP. **(A1)** *P. intermedia* with untreated, as control; **(A2)** *P. intermedia* treated with MIC; **(A3)** *P. intermedia* treated with MBC; **(B1)** *P. gingivalis* with untreated, as control; **(B2)** *P. gingivalis* treated with MIC; **(B3)** *P. gingivalis* treated with MBC; **(C1)** *F. nucleatum* with untreated, as control; **(C2)** *F. nucleatum* treated with MIC; **(C3)** *F. nucleatum* treated with MBC. MIC, minimum inhibition concentration; MBC, minimum bactericide concentration.

Transmission electron microscopic analysis was performed on the bacterial cells treated with different concentrations of YAP (control, MIC, and MBC). As shown in Figure 3, untreated bacterial cells (Figures 3A1,B1,C1) showed that the cells have complete cell walls and membranes, and homogeneous intracellular constituents. After treatment, the TEM images in Figures 3A2,B2,C2 showed the surface of bacterial cells treated with YAP at the MIC level. Some part of the cell wall became blurred. The cell walls and membranes were separated, and the intracellular constituents were inhomogeneous. Furthermore, the cells treated with YAP at the MBC level (Figures 3A3,B3,C3) exhibited that the cell wall was ruptured and the cytoplasmic content was leaked from the cell. These observations of TEM further confirmed that YAP could damage the cell walls and membranes and alter the cell intracellular microstructure.

### Effect of YAP on Cell Membrane Integrity

**FIGURE 4:**
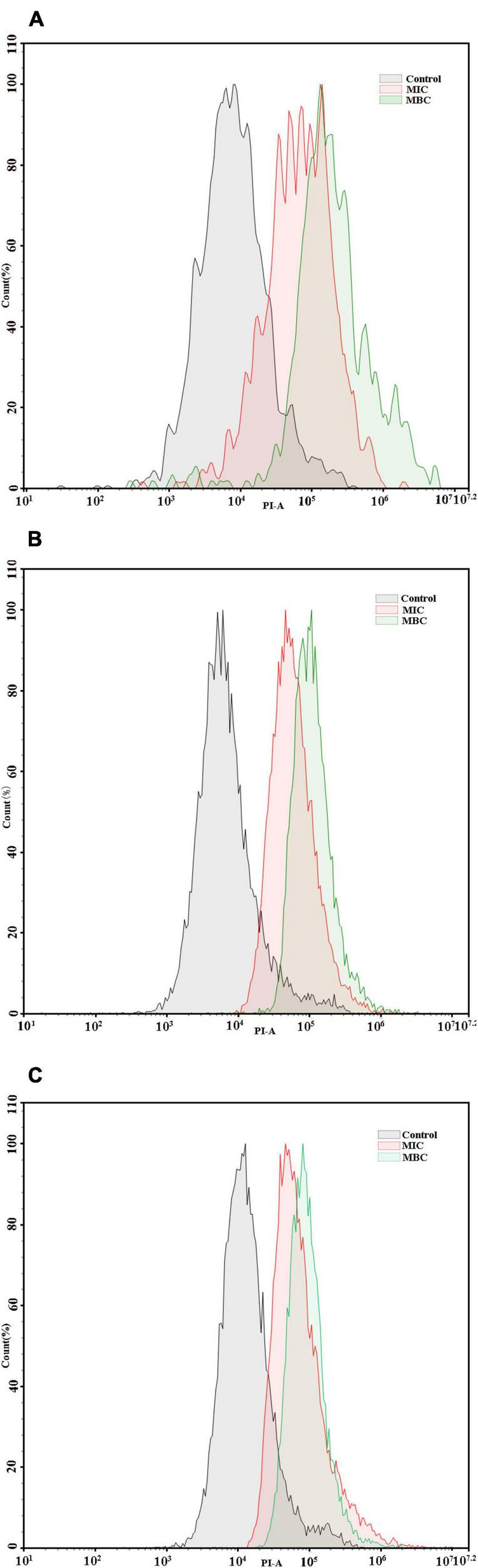
Flow cytometric analysis. Data acquisition was set to 30 μl for each experiment. **(A)** *F. nucleatum*; **(B)** *P. gingivalis*; **(C)** *P. intermedia.* MIC, minimum inhibition concentration; MBC, minimum bactericide concentration.

The integrity of cell membranes was analyzed by flow cytometry with the fluorescent probe PI (Figure 4). When the integrity of the cell membranes was damaged, the fluorescence intensity of the bacterial cells may increase. The percentages of *F. nucleatum*, *P. gingivalis*, and *P. intermedia* cells with PI fluorescence in the MIC group were 53.47, 52.91, and 57.14%, respectively, while the percentages of bacterial cells with PI fluorescence in the MBC group were 76.92, 94.51, and 78.77%, respectively. The flow cytometry results showed that the cells with the integrity membrane dramatically reduced with the increase in YAP concentrations (*p* < 0.05).

### Effect of YAP on the Release of Proteins and Nucleic Acids

**FIGURE 5:**
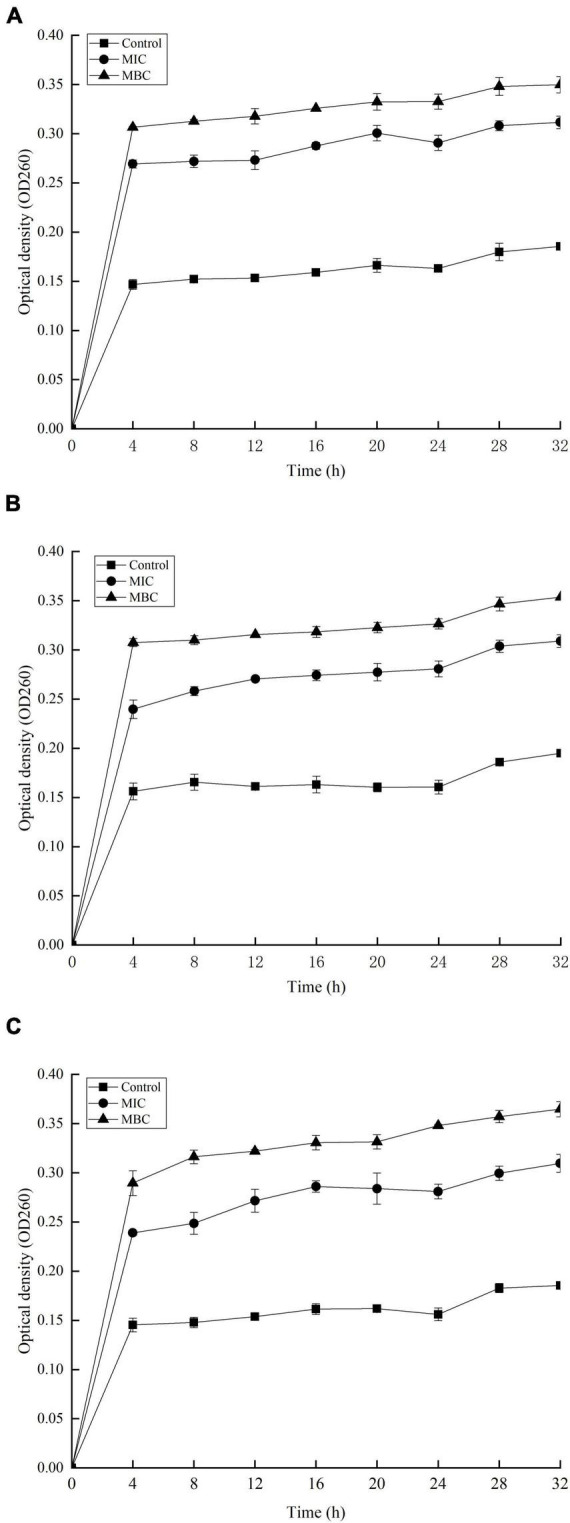
Release of 260 nm absorbing material from **(A)** *F. nucleatum*; **(B)** *P. gingivalis*; and **(C)** *P. intermedia* treated with YAP for 32 h. MIC, minimum inhibition concentration; MBC, minimum bactericide concentration.

**FIGURE 6:**
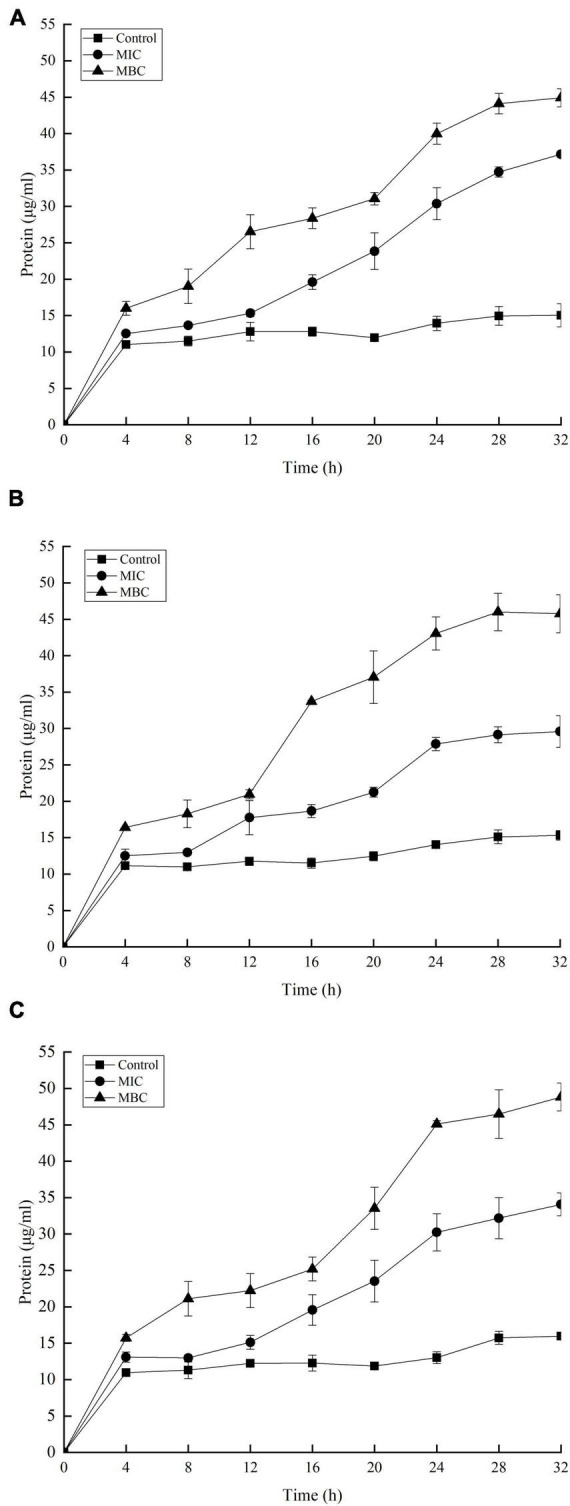
Release of protein-absorbing material from **(A)** *F. nucleatum*; **(B)** *P. gingivalis*; and **(C)** *P. intermedia* treated with YAP for 32 h. MIC, minimum inhibition concentration; MBC, minimum bactericide concentration.

The release of proteins and nucleic acids into the cell suspension was studied to further explore the effect of YAP on the bacterial cells. The OD_260__nm_ values showed the release of nucleic acids into the suspension of the bacterial cells with different YAP concentration treatments, as shown in Figure 5. The OD_260__nm_ values of the three bacteria after YAP treatment at the MBC level were higher than those after the MIC treatment, which suggested that the release of nucleic acids significantly increased with the increase in YAP concentrations (*p* < 0.05). Compared to the untreated group, the OD_260__nm_ values of the three bacteria treated with YAP at both MIC and MBC levels significantly increased from 0 to 4 h (*p* < 0.05). However, the OD_260__nm_ values of the three bacteria treated with MIC and MBC of YAP steadily increased for the next tested hours, and its increasing rate was obviously lower than that for the first 4 h. Figure 6 shows the release of protein content of three bacterial cells from 0 to 32 h. When the three bacterial cells were treated with YAP at the MBC level, the released protein content of three bacterial cells was significantly higher than that at the MIC level. Additionally, compared to the untreated group, the release of protein content remarkably increased from 0 to 4 h for three bacterial cells after YAP treatment with MBC and MIC levels, while the release of protein content of three bacterial cells after YAP treatment also increased from 4 to 24 h, but the increasing rate was lower than that from 0 to 4 h. For the last 8 h, the release of protein content of three bacterial cells remained stable, especially for *P. gingivalis* and *P. intermedia.*

### Effect of YAP on Membrane Potential

**FIGURE 7:**
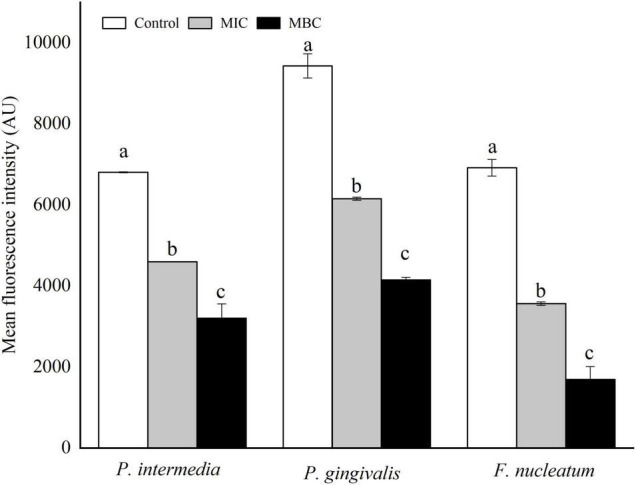
Membrane potentials of *P. intermedia*, *P. gingivalis*, and *F. nucleatum* treated with different concentrations of YAP. MIC, minimum inhibition concentration; MBC, minimum bactericide concentration.

Figure 7 shows the changes in membrane potential (MP) of three bacterial cells after YAP treatment. Compared with the untreated group, the MFI values of *P. intermedia*, *P. gingivalis*, and *F. nucleatum* decreased by 32.48, 34.74, and 48.47%, respectively, after YAP treatment at the MIC level, while the MFI values reduced by 52.97, 56.04, and 75.57%, respectively, after YAP treatment at the MBC level. The results indicated that the MP of three bacterial cells significantly reduced with the increase in YAP concentration (*p* < 0.05). The decrease in the cell MP means the depolarization of the cell membrane, which could eventually lead to irregular cell metabolism and cause cell death (Hamilton et al., 2021).

### Principal Component Analysis

**FIGURE 8:**
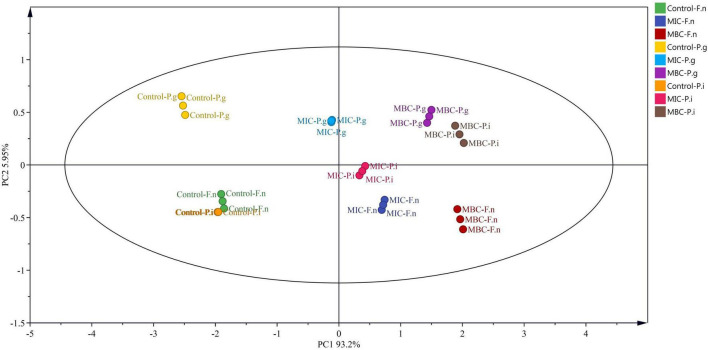
Loadings of *P. intermedia*, *P. gingivalis*, and *F. nucleatum* treated with different concentrations of YAP on the first and second principal components. F.n, *F. nucleatum*; P.g, *P. gingivalis*; P.i, *P. intermedia*; MIC, minimum inhibition concentration; MBC, minimum bactericide concentration.

The relationships between the release of proteins and nucleic acids and MP of halitosis-related bacteria treated with different concentrations of YAP were analyzed by principal component analysis (Figure 8) (PCA) (Pu et al., 2020). The first component (PC1) and the second component (PC2) accounted for 99.15% of the total variance, which indicated that the first two principal components could distinguish the bacteria treated with different concentrations of YAP. Along with the direction of PC1, the control group is mainly distributed in the negative half axis of PC1, while the group treated with MIC and MBC is mainly distributed on the positive half axis, indicating that there is a significant difference between the groups treated with YAP and the control group. Surprisingly, according to the PC1 direction, the bacterial groups treated with YAP at the MIC level plotted was between the control and bacterial groups treated with YAP at the MBC level; this may be because under the treatment of MIC and MBC, the MP of bacterial cells was significantly reduced, and the release of proteins and nucleic acids was significantly increased. It also indicates that the effect of YAP content on the bacteria-related halitosis was significant. Along with the direction of PC2, the groups of *P. gingivalis* treated with different YAP concentrations were distributed at the positive half axis of PC2, while the groups of *F. nucleatum* were distributed at the negative half axis of PC2, which may be due to the different sensitivities to the pH of culture medium (Takahashl et al., 2010).

## Discussion

Although many literatures are focused on the antibacterial effects of apple polyphenols, there are few literatures on thinned young apple polyphenols. We extracted and purified polyphenols from ‘Fuji’ thinned young apples according to the methods of our previous publication (Gong et al., 2020) and analyzed the extracted YAP by HPLC. The higher contents of individual phenols in thinned young apples were chlorogenic acid and phlorizin. It was found that chlorogenic acid and phloretin/phlorizin, as natural antibacterial agents, are widely used in pharmaceutical products (Li et al., 2014; Zhang et al., 2016). However, the phenol extract from Golden Delicious apples by ethyl acetate had a lower antibacterial activity against *Staphylococcus aureus* and *Escherichia coli* than the phlorizin and phloretin standards (Zhang et al., 2016), which may be due to the fact that the polyphenols from apples were usually conjugated with glycosides and reduced the antibacterial activity (Cao et al., 2009). The relative position of the hydroxyl group in the phenolic compounds and the types of alkyl substituents incorporated into the non-phenolic ring structure could also affect the antibacterial activity (Dorman and Deans, 2000). In our study, the YAP showed a certain antibacterial activity, but the antibacterial activity was lower than other natural phenolic extracts, such as tea polyphenols and tart cherry phenolic extract (Lagha et al., 2017, 2021). In order to improve the antibacterial activity of YAP, the structure of YAP should be further studied and modified in the future.

*Fusobacterium nucleatum*, *P. gingivalis*, and *P. intermedius* have been strongly associated with periodontal lesions as well as halitosis (Awano et al., 2002; Tonetti et al., 2013). Morphological alterations could intuitively reflect the antibacterial effects of YAP against the halitosis-related bacteria. In the present study, we investigated the influence of different concentrations of YAP (control, MIC, and MBC) on morphological alterations in bacterial cells through SEM and TEM analyses. After YAP treatment, the SEM analyses observed that the membrane surface was coarse and wrinkles and pores occurred, which was consistent with other research about the extracted phenols of fruits, such as hawthorn and wild blueberry (Ben et al., 2015; Zhang et al., 2020). The TEM analyses observed that the cell walls and membranes were separated and eventually ruptured and the cell contents were leaked, which were irreversible. The morphological alterations of bacteria cells may be because the phenolic acid could change the hydrophobicity of the cell membrane, resulting in irreversible changes in intracellular components (Borges et al., 2013; Alshuniaber et al., 2020).

In prokaryotes, the cell membrane is not only related to the energy conversion but also related to nutrient processing, the synthesis of structural macromolecules, and the secretion of many enzymes required for life (Yuroff et al., 2003; Silhavy, 2016). Thus, the integrity of the cell membrane is essential for cell growth. The flow cytometer is used to determine the integrity of the cell membrane (Cao et al., 2009), and the release of large molecules including nucleic acids and proteins into the cell suspension could be further evaluated to determine whether the cell membrane integrity is damaged (Diao et al., 2014; Zhou et al., 2020). Our results indicated that the integrity of the cell membrane was destroyed and the leakage of proteins and nucleic acids into the cell suspension increased after YAP treatment. These phenomena provided evidence that the bacterial cell membranes are damaged, which agreed with the antibacterial activity of tea polyphenols (Yi et al., 2010). Lou et al. (2011) found that the negatively charged phenol compounds may be attached to the outer membrane of bacteria by the electrostatic interaction and destroy the outer membrane. Additionally, the study has shown that the electronegative phenol compounds could interfere with the biological process by the electron transfer and react with the nitrogen components such as nucleic acids and proteins (Dorman and Deans, 2000).

The MP is one of the most important parameters of bacterial cells. It is related to cellular antibiotic intake and bactericidal effect (Bajpai et al., 2013). A lot of information could be obtained by monitoring the MP of a cell. When the cell membrane is damaged, depolarization and hyperpolarization will be shown (Comas and Vives-Rego, 1997). Depolarization and hyperpolarization were mainly caused by the changing of the ion concentration in cells, which could damage the functions of the cell membrane (Eisenberg et al., 1982). Therefore, the MP could be used to determine whether the cell membrane of bacteria is damaged. In our study, the MP of bacteria-related halitosis significantly reduced after YAP treatment. These results suggested that YAP could effectively induce the depolarization of the bacterial cell membrane and damage the cell membrane, resulting in abnormal metabolism of bacterial cells. Studies found that the halitosis-related bacteria could be depolarized by quinoa saponins, which is consistent with our research results (Sun et al., 2019). Lou et al. (2011) reported that a large amount of K^+^ was released to the cell suspension of *Shigella dysenteriae* and *Streptococcus pneumoniae* after chlorogenic acid treatment, which was due to the fact that chlorogenic acid changed the MP and damaged the cell membrane. However, the effect of YAP on the ions released from halitosis-related bacterial cells still needs to be further studied in the future.

The content of polyphenols of thinned young apples is about 10 times compared with ripe apples (Hiroshi et al., 2005). In our study, we explored the antibacterial effect and antibacterial mechanism of YAP on halitosis-related bacterial. The results indicated that YAP could inhibit *P. gingivalis*, *P. intermedius*, and *F. nucleatum* and alter the morphology of bacterial cells and the integrity of cell membranes. YAP had potential roles for curing oral odor induced by bacteria. However, the polyphenol was always attached with other large molecules such as protein and polysaccharide in food systems. Our future work should aim at investigating the influence of the protein, organic acid, and polysaccharide combined with the YAP, especially chlorogenic acid and phlorizin, on the antibacterial activity against halitosis-related bacteria and clarifying the antibacterial mechanism.

## Conclusion

This study clearly showed that the phenolic extract from ‘Fuji’ has an inhibitory effect on halitosis-related bacteria, including *P. gingivalis*, *P. intermedius*, and *F. nucleatum*. The outer wall of the bacterial cells treated with YAP showed obvious wrinkles and holes, while in the internal microstructure, the cell wall and cell membrane were separated, blurred, and even disappeared. The release of proteins and nucleic acids into the cell suspension significantly increased with the increase in YAP concentration treatment. The MP of three bacterial cells treated with YAP significantly reduced. These results revealed that YAP could destroy the integrity and permeability of the cell membrane, resulting in the cell death of bacteria related with halitosis. This research could open up new areas for the application of thinned young apples and provided new antibacterial agents for halitosis.

## Data Availability Statement

The original contributions presented in the study are included in the article/supplementary material, further inquiries can be directed to the corresponding authors.

## Author Contributions

TL: conceptualization, methodology, validation, formal analysis, investigation, data curation, writing—original draft, and visualization. HS: visualization and investigation. FW: methodology and software. XZ and PZ: methodology. YY: funding acquisition, supervision, resources, and writing—review and editing. YG: funding acquisition and resources. All authors contributed to the article and approved the submitted version.

## Conflict of Interest

The authors declare that the research was conducted in the absence of any commercial or financial relationships that could be construed as a potential conflict of interest.

## Publisher’s Note

All claims expressed in this article are solely those of the authors and do not necessarily represent those of their affiliated organizations, or those of the publisher, the editors and the reviewers. Any product that may be evaluated in this article, or claim that may be made by its manufacturer, is not guaranteed or endorsed by the publisher.

## Abbreviations

YAP: thinned-young apple polyphenols
*F. nucleatum*: *Fusobacterium nucleatum*
*P. gingivalis*: *Porphyromonas gingivalis*
*P. intermedius*: *Prevotella intermedius*
SEM: scanning electron microscopy
TEM: transmission electron microscope
MIC: minimum inhibitory concentration
MBC: minimum bactericidal concentration
CFU/mL: colony-forming unit per milliliter
PI: propidium iodide
MFI: mean fluorescence intensity
PCA: principal component
OD_260__nm_: optimal density at 260 nm
MP: membrane potential
PC1: the first component
PC2: the second component.

## References

[B1] Alshuniaber M. A. Krishnamoorthy R. Alqhtani W. H. (2020). Antimicrobial activity of polyphenolic compounds from Spirulina against food-borne bacterial pathogens. *Saudi. J. Biol. Sci.* 28 459–464. 10.1016/j.sjbs.2020.10.029 33424328 PMC7783674

[B2] Armstrong B. L. Sensat M. L. Stoltenberg J. L. (2010). Halitosis: a review of current literature. *Int. J. Dent. Hyg*. 84 65–74. 10.4274/meandros.68077 20359417

[B3] Awano S. Gohara K. Kurihara E. Ansai T. Takehara T. (2002). The relationship between the presence of periodontopathogenic bacteria in saliva and halitosis. *Int. Dent. J.* 52 212–216. 10.1002/j.1875-595X.2002.tb00927.x 12090455

[B4] Bajpai V. K. Sharma A. Baek K. H. (2013). Antibacterial mode of action of Cudrania tricuspidata fruit essential oil, affecting membrane permeability and surface characteristics of food-borne pathogens. *Food Control* 32 582–590. 10.1016/j.foodcont.2013.01.032

[B5] Ben L. A. Dudonné S. Desjardins Y. Grenier D. (2015). Wild Blueberry (Vaccinium angustifolium Ait.) Polyphenols Target *Fusobacterium nucleatum* and the Host Inflammatory Response: potential Innovative Molecules for Treating Periodontal Diseases. *J. Agric. Food. Chem.* 63 6999–7008. 10.1021/acs.jafc.5b01525 26207764

[B6] Borges A. Ferreira C. Saavedra M. J. Simes M. (2013). Antibacterial Activity and Mode of Action of Ferulic and Gallic Acids Against Pathogenic Bacteria. *Larchmont. N.Y.* 19 256–265. 10.1089/mdr.2012.0244 23480526

[B7] Bradford M. M. (1976). A rapid and sensitive method for the quantitation of microgram quantities of protein utilizing the principle of protein-dye binding. *Anal. Biochem.* 72 248–254. 10.1016/0003-2697(76)90527-90523 942051

[B8] Cao X. L. Wang C. Pei H. R. Sun B. G. (2009). Separation and identification of polyphenols in apple pomace by high-speed counter-current chromatography and high-performance liquid chromatography coupled with mass spectrometry. *J. Chromatogr A*. 1216 4268–4274. 10.1016/j.chroma.2009.01.046 19203755

[B9] Chen L. Yang X. Liu R. Liu L. Zhao D. Liu J. (2017). Thinned young apple polysaccharide improves hepatic metabolic disorder in high-fat diet-induced obese mice by activating mitochondrial respiratory functions. *J. Funct. Foods* 33 396–407. 10.1016/j.jff.2017.03.055

[B10] Chen L. Y. Cheng C. W. Liang J. Y. (2015). Effect of esterification condensation on the folin-ciocalteu method for the quantitative measurement of total phenols. *Food Chem*. 170 10–15. 10.1016/j.foodchem.2014.08.038 25306311

[B11] Chen W. Guo Y. Zhang J. Zhang X. Meng Y. (2015). Effect of Different Drying Processes on the Physicochemical and Antioxidant Properties of Thinned Young Apple. *Int. J. Food. Eng*. 11 207–219. 10.1515/ijfe-2014-0211

[B12] Comas J. Vives-Rego J. (1997). Assessement of the effects of gramicidin, formaldehyde, andsurfactants on *Escherichia coli* by flow cytometry using nucleic acid and membrane potential dyes. *Cytometry* 29 58–64. 10.1002/(sici)1097-0320(19970901)29:1<58::aid-cyto6<3.0.co;2-9 9298812

[B13] Cortelli J. R. Silva B. M. D. Westphal M. A. (2008). Halitosis: a review of associated factors and therapeutic approach. *Braz. Oral. Res.* 22 44–54. 10.1590/S1806-83242008000500007 19838550

[B14] Diao W. R. Hu Q. P. Zhang H. Xu J. G. (2014). Chemical composition, antibacterial activity and mechanism of action of essential oil from seeds of fennel (Foeniculum vulgare mill.). *Food Control* 35 109–116. 10.1016/j.foodcont

[B15] Dorman H. J. Deans S. G. (2000). Antimicrobial agents from plants: antibacterial activity of plant volatile oils. *J. Appl. Microbiol*. 88 308–316. 10.1046/j.1365-2672.2000.00969.x 10736000

[B16] Dou J. Meng Y. Liu L. Li J. Ren D. Guo Y. (2015). Purification, characterization and antioxidant activities of polysaccharides from thinned-young apple. *Int. J. Biol. Macromol*. 72 31–40. 10.1016/j.ijbiomac.2014.07.053 25109456

[B17] Eisenberg E. S. Mandel L. J. Kaback H. R. Miller M. H. (1982). Membrane potential and gentamicin uptake in *Staphylococcus aureus*. *P. Natl. Acad. Sci. U.S.A.* 79 6693–6697. 10.1073/pnas.79.21.6693 6959147 PMC347195

[B18] Gong T. Yang X. Bai F. T. Li D. Zhao P. T. Sun L. J. (2020). Young apple polyphenols as natural α-glucosidase inhibitors: in vitro and in silico studies. *Bioorg. Chem.* 96:103625. 10.1016/j.bioorg.2020.103625 32028059

[B19] Hamilton J. Brustovetsky T. Brustovetsky N. (2021). The effect of mitochondrial calcium uniporter and cyclophilin D knockout on resistance of brain mitochondria to Ca^2+^ -induced damage. *J. Biol. Chem.* 296:100669. 10.1016/j.jbc.2021.100669 33864812 PMC8131324

[B20] Hiroshi A. Yuji S. Takahiro W. Megumi H. N. Yasuo Y. Toshihiko S. (2005). Dietary unripe apple polyphenol inhibits the development of food allergies in murine models. *Febs. Lett.* 579 4485–4491. 10.1016/j.febslet.2005.07.019 16081068

[B21] Hou Y. Gong T. Zhang J. Yang X. Guo Y. (2019). Structural characterization and emulsifying properties of thinned-young apples polysaccharides. *Biochem*. *Bioph*. *Res*. *Co*. 516 1175–1182. 10.1016/j.bbrc.2019.07.019 31296384

[B22] Joda J. Olukoju O. (2013). Halitosis amongst students in tertiary institutions in lagos state. *Afr. Health. Sci*. 12 473–478. 10.4314/ahs.v12i4.12 23515705 PMC3598288

[B23] Lagha A. B. Haas B. Grenier D. (2017). Tea polyphenols inhibit the growth and virulence properties of *Fusobacterium nucleatum*. *Sci. Rep.* 7:44815. 10.1038/srep44815 28322293 PMC5359671

[B24] Lagha A. B. Lebel G. Grenier D. (2020). Tart cherry (Prunus cerasus L.) fractions inhibit biofilm formation and adherence properties of oral pathogens and enhance oral epithelial barrier function. *Phytother. Res.* 34 886–895. 10.1002/ptr.6574 31846135

[B25] Lagha A. B. Pellerin G. Vaillancourt K. Grenier D. (2021). Effects of a tart cherry (Prunus cerasus L.) phenolic extract on *Porphyromonas gingivalis* and its ability to impair the oral epithelial barrier. *PLoS One* 16:e0246194. 10.1371/journal.pone.0246194 33497417 PMC7837497

[B26] Lau P. Meethal C. Middleton M. Clark M. Darby I. (2019). ‘Say Ahhh’: what do dentists, general medical practitioners and community pharmacists do about halitosis? *Int. Dent. J*. 69 311–320. 10.1111/idj.12458 30565208 PMC9379005

[B27] Li G. Qiao M. Guo Y. Wang X. Xia X. (2014). Effect of subinhibitory concentrations of chlorogenic acid on reducing the virulence factor production by *Staphylococcus aureus*. *Foodborne Pathog. Dis.* 11 677–683. 10.1089/fpd.2013.1731 24905974

[B28] Li M. Guo J. Xu C. Lei Y. Li J. (2018). Identifying climatic factors and circulation indices related to apple yield variation in main production areas of china. *Glob. Ecol. Conserv*. 16:e00478. 10.1016/j.gecco.2018.e00478

[B29] Lou Z. Wang H. Song Z. Ma C. Wang Z. (2011). Antibacterial Activity and Mechanism of Action of Chlorogenic Acid. *J. Food Sci.* 76 M398–M403. 10.1111/j.1750-3841.2011.02213.x 22417510

[B30] Miller N. J. Rice-Evans C. A. (1997). The relative contributions of ascorbic acid and phenolic antioxidants to the total antioxidant activity of orange and apple fruit juices and blackcurrant drink. *Food Chem*. 60 331–337. 10.1016/S0308-8146(96)00339-1

[B31] Nicolas J. J. Richard-Forget F. C. Goupy P. M. Amiot M. J. Aubert S. Y. (1994). Enzymatic browning reactions in apple and apple products. *Crit*. *Rev*. *Food Sci*. 34 109–157. 10.1080/10408399409527653 8011143

[B32] Nijole S. Aiste J. Lina R. Asmaa A. Andrea C. Lia R. (2018). Efficacy of proanthocyanidins from pelargonium sidoides root extract in reducing *p. gingivalis* viability while preserving oral commensal *s. salivarius*. *Materials* 11:1499. 10.3390/ma11091499 30135370 PMC6164244

[B33] Nisar T. Wang Z. C. Alim A. Iqbal M. Yang X. Sun L. J. (2019). Citrus pectin films enriched with thinned young apple polyphenols for potential use as bio-based active packaging. *CyTA J. Food* 17 695–705. 10.1080/19476337.2019.1640798

[B34] Peruzzo D. C. Jandiroba P. F. C. B. Filho G. D. R. N. (2007). Use of 0.1% chlorine dioxide to inhibit the formation of morning volatile sulphur compounds (VSC). *Braz. Oral. Res.* 21 70–74. 10.1590/S1806-83242007000100012 17384858

[B35] Pu D. Duan W. Huang Y. Zhang Y. Sun B. Ren F. (2020). Characterization of the key odorants contributing to retronasal olfaction during bread consumption. *Food Chem*. 318:126520. 10.1016/j.foodchem.2020.126520 32155563

[B36] Sanz M. Roldán S. Herrera D. (2001). Fundamentals of breath malodour. *J. Contemp. Dent. Pract*. 2 1–17. 10.5005/jcdp-2-4-22 12167916

[B37] Silhavy T. J. (2016). Classic Spotlight: gram-Negative Bacteria Have Two Membranes. *J. Bacteriol.* 198:201. 10.1128/JB.00599-15 26715373 PMC4751781

[B38] Sun X. Y. Yang X. S. Xue P. Zhang Z. G. Ren G. X. (2019). Improved antibacterial effects of alkali-transformed saponin from quinoa husks against halitosis-related bacteria. *BMC. Complem. Altern. M.* 19:46. 10.1186/s12906-019-2455-2 30755185 PMC6373059

[B39] Takahashl N. Saito K. Schachtele C. F. Yamada T. (2010). Acid tolerance and acid−neutralizing activity of *Porphyromonas gingivalis*, *Prevotella intermedia* and *Fusobacterium nucleatum*. *Mol. Oral. Microbiol.* 12 323–328. 10.1111/j.1399-302X.1997.tb00733.x 9573805

[B40] Tonetti M. S. Van Dyke T. E. Working group 1 of the joint EFP/AAP workshop (2013). Periodontitis and atherosclerotic cardiovascular disease: consensus report of the Joint EFP/AAP Workshop on Periodontitis and Systemic Diseases. *J. Clin. Periodontol.* 40 S24–S29. 10.1111/jcpe.12089 29537596

[B41] Wang J. Y. Zhang W. J. Tang C. E. Xiao J. Xie B. J. Sun Z. D. (2018). Synergistic effect of B-type oligomeric procyanidins from lotus seedpod in combination with water-soluble Poria cocos polysaccharides against *E. coli* and mechanism. *J. Funct. Foods* 48 134–143. 10.1016/j.jff.2018.07.015

[B42] Wang L. Yang X. Yu X. Yao Y. Ren G. (2013). Evaluation of antibacterial and anti-inflammatory activities of less polar ginsenosides produced from polar ginsenosides by heat-transformation. *J. Agr. Food Chem.* 61 12274–12282. 10.1021/jf404461q 24289140

[B43] Wang L. Yang X. Z. Hu D. Y. (2002). Anti-halitosis effect of radix scutellariae and tea polyphenol. *Int. Dent. J*. 52 212–216. 10.1016/S1005-8885(07)60038-712090455

[B44] Wang X. Wang J. Wei L. Hu C. Y. Meng Y. H. (2019). Apple phlorizin oxidation product 2 inhibits proliferation and differentiation of 3t3-l1 preadipocytes. *J. Funct. Food*. 62:103525. 10.1016/j.jff.2019.103525

[B45] Yi S. M. Zhu J. L. Fu L. L. Li J. R. (2010). Tea polyphenols inhibit *Pseudomonas aeruginosa* through damage to the cell membrane. *Int. J. Food Microbiol.* 144 111–117. 10.1016/j.ijfoodmicro 20884071

[B46] Yuan L. Lijun S. Chen W. Q. Meng Y. H. Guo Y. R. (2016). ctions between polyphenols in thinned young apples and porcine pancreatic alpha-amylase: inhibition, detailed kinetics and fluorescence quenching. *Food Chem*. 208 51–60. 10.1016/j.foodchem.2016.03.093 27132823

[B47] Yuroff A. S. Sabat G. Hickey W. J. (2003). Transporter-Mediated Uptake of 2-Chloro- and 2-Hydroxybenzoate by *Pseudomonas* huttiensis Strain D1. *Appl. Environ. Microb.* 69 7401–7408. 10.1128/AEM PMC309881 14660391

[B48] Zhang J. Chen W. Shuai L. Xue Z. Guo Y. (2017). Antibacterial activity and preservative properties of thinned young apples extracts for fish flesh. *J. Food Process Pres.* 42:e13435. 10.1111/jfpp.13435

[B49] Zhang L. L. Zhang L. F. Xu J. G. (2020). Chemical composition, antibacterial activity and action mechanism of different extracts from hawthorn (Crataegus pinnatifida Bge.). *Sci. Rep. U.K.* 10:8876. 10.1038/s41598-020-65802-7 32483369 PMC7264281

[B50] Zhang T. Wei X. Miao Z. Hassan H. Song Y. Fan M. (2016). Screening for antioxidant and antibacterial activities of phenolics from Golden Delicious apple pomace. *Chem. Cent. J.* 10:47. 10.1186/s13065-016-0195-7 27486478 PMC4970275

[B51] Zhou Y. Yao Q. Zhang T. Chen X. Cheng Y. (2020). Antibacterial activity and mechanism of green tea polysaccharide conjugates against *Escherichia coli*. *Ind. Crop Prod.* 152:112464. 10.1016/j.indcrop.2020.112464

